# Suitability of Single-Nucleotide Polymorphism Arrays Versus Genotyping-By-Sequencing for Genebank Genomics in Wheat

**DOI:** 10.3389/fpls.2020.00042

**Published:** 2020-02-14

**Authors:** Jianting Chu, Yusheng Zhao, Sebastian Beier, Albert W. Schulthess, Nils Stein, Norman Philipp, Marion S. Röder, Jochen C. Reif

**Affiliations:** ^1^Department of Breeding Research, Leibniz Institute of Plant Genetics and Crop Plant Research (IPK), Seeland, Germany; ^2^Department of Genebank, Leibniz Institute of Plant Genetics and Crop Plant Research (IPK), Seeland, Germany; ^3^Faculty of Sciences III - Agricultural and Nutritional Sciences, Earth Sciences and Computer Science, Martin-Luther-University Halle-Wittenberg, Halle/Saale, Germany

**Keywords:** single-nucleotide polymorphism (SNPs), genotyping-by-sequencing (GBS), simple sequence repeats (SSR), genebank genomics, molecular diversity, genome-wide prediction, wheat

## Abstract

Genebank genomics promises to unlock valuable diversity for plant breeding but first, one key question is which marker system is most suitable to fingerprint entire genebank collections. Using wheat as model species, we tested for the presence of an ascertainment bias and investigated its impact on estimates of genetic diversity and prediction ability obtained using three marker platforms: simple sequence repeat (SSR), genotyping-by-sequencing (GBS), and array-based SNP markers. We used a panel of 378 winter wheat genotypes including 190 elite lines and 188 plant genetic resources (PGR), which were phenotyped in multi-environmental trials for grain yield and plant height. We observed an ascertainment bias for the array-based SNP markers, which led to an underestimation of the molecular diversity within the population of PGR. In contrast, the marker system played only a minor role for the overall picture of the population structure and precision of genome-wide predictions. Interestingly, we found that rare markers contributed substantially to the prediction ability. This combined with the expectation that valuable novel diversity is most likely rare suggests that markers with minor allele frequency deserve careful consideration in the design of a pre-breeding program.

## Introduction

Global agricultural production must be increased by 60% compared to 2005–2007 levels in order to supply an estimated world population of 9 billion in 2050 (Ray et al., 2013; FAO, 2017). The annual yield increases for the four main crops (wheat, corn, rice, and soybean) are about 0.9%–1.6%, which is far below the required one (Ray et al., 2013). It is becoming increasingly difficult to meet this rising global demand as arable land and water become scarcer, average living standards rise, and investments to increase agricultural productivity grow slowly (Fischer et al., 2014; Laidig et al., 2014). Wheat breeding is a viable and sustainable solution for increasing grain yield and improving yield stability (Borlaug, 1968; Voss-Fels et al., 2019).

The success of wheat breeding strongly depends on the availability of a valuable diversity within breeding populations (Jannink et al., 2010; Rufo et al., 2019). The effective population size in European wheat breeding populations is small with an estimated value of ~30 individuals (He et al., 2017). Therefore, the extension of the genetic diversity of elite wheat breeding pools through the introgression of valuable variation is crucial for increasing the grain yield potential. Moreover, the systematic genotyping of collections was proposed as a first step toward developing new ways and approaches to unlock wheat genetic resources for breeding (Mascher et al., 2019). Genotyping of plant genetic resources (PGRs) was performed for some important crops such as barley (Milner et al., 2019), maize (Romay et al., 2013), and rice (Wang et al., 2018). As far as wheat is concerned, many efforts have focused on how genomic technologies can be used to genotype PGRs (Rasheed et al., 2018). For example, the global landrace collection “Watkins” was genotyped with 41 simple sequence repeat (SSR) markers for 826 landraces from 32 countries (Wingen et al., 2014). A collection of 295 accessions including 136 landraces from 25 countries from the Australian Grains genebank was fingerprinted by genotyping-by-sequencing (GBS) and Diversity Arrays Technology (DArT-seq) (Riaz et al., 2017). An 820k Axiom single-nucleotide polymorphism (SNP) array as well as a 35k subset were developed by genotyping 43 bread wheat lines including their wild accessories (Winfield et al., 2016; King et al., 2017). The GBS platform was also used for genotyping “Creole” landraces conserved in CIMMYT's genebank (Vikram et al., 2016), a sample of 62 diverse wheat lines including 26 landraces (Jordan et al., 2015), a set of 1,143 accessions of *Aegilops tauschii* (Singh et al., 2019) and a set of 1,423 spring bread wheat germplasm including 561 landrace accessions (Sehgal et al., 2015). These recent works present the potential of introducing exotic alleles present in these PGRs to improve elite wheat lines. In this sense, the genomic data not only allow to estimate the neutral molecular diversity of genetic resources as compared to that of elite lines (He et al., 2019) but also to combine it with phenotypic information in order to find novel valuable functional genetic variation, i.e. genes/alleles/haplotypes (e.g., Milner et al., 2019) or to build up genome-wide prediction models to select promising candidates for (pre)breeding (Yu et al., 2016). Whole-genome sequencing of entire collections is currently not affordable in large-genome species such as wheat and therefore attempts have been mainly focused on cost-effective genotyping platforms (Milner et al., 2019). Several marker platforms have been developed in wheat in the past (Elbasyoni et al., 2018). SSR markers (Röder et al., 1995; Röder et al., 1998) were replaced by diversity array technology (DArT markers; Wenzl et al., 2004), GBS (Elshire et al., 2011; Poland et al., 2012), and array platforms for scoring SNPs (Cavanagh et al., 2013; Wang et al., 2014; Winfield et al., 2016). The disadvantage of most cost-efficient genotyping platforms in contrast to whole-genome sequencing is that an ascertainment bias can be introduced by designing the marker platforms using a limited set of individuals (Clark et al., 2005). This has been described for instance in maize (e.g., Frascaroli et al., 2013). An ascertainment bias can impact the estimates of the diversity within populations but seems to be of minor relevance for the estimates of the overall population structure (Heslot et al., 2013; Alipour et al., 2017; Eltaher et al., 2018; Bhatta et al., 2018) or further downstream applications such as genome-wide predictions (Heslot et al., 2013; Jiang et al., 2015; Elbasyoni et al., 2018). For wheat, only a few studies have compared the accuracy of genome-wide prediction between SSR and SNP array markers (e.g., Jiang et al., 2015), between GBS and DArT markers (e.g., Heslot et al., 2013), and between GBS and SNP array markers (e.g., Elbasyoni et al., 2018). The results heavily depend on the underlying germplasm, while studies on the relevance of an ascertainment bias on diversity estimates and genome-wide predictions in wheat genetic resources are rare. Furthermore, it is also promising to test whether genetic information from different marker platforms is complementary and whether their integrated use can boost prediction accuracies.

The objectives of our study were to 1) compare the relevance of an ascertainment bias on the genetic diversity estimated by SSR, GBS, and SNP array markers in a wheat population comprising PGRs and European elite lines, 2) contrast the prediction ability obtained using the three marker platforms, and 3) investigate the potential and limits of genome-wide prediction models exploiting the complementarity of different marker platforms.

## Materials and Methods

### Genotyping and Population Genetic Analyses

We fingerprinted 378 winter wheat (*Triticum aestivum* L.) genotypes: 190 lines represent the elite breeding pool exploited in Europe (Elite) and 188 genotypes represent a random sample of PGRs maintained at the genebank of the IPK Gatersleben, Germany. Details on the plant material have already been published (Philipp et al., 2018). The 378 wheat lines were characterized using (1) an Infinium 90,000 SNP array for 174 genotypes out of 571 samples (Wang et al., 2014) and a derived Infinium 15,000 SNP array for 204 genotypes out of 782 samples (Boeven et al., 2019), (2) GBS (Wendler et al., 2014), and (3) 19 SSR markers (Plaschke et al., 1995; Röder et al., 1995; Röder et al., 1998). The 90,000 SNP array data were used from a previously published study (Zanke et al., 2014a; Zanke et al., 2014b; Zanke et al., 2015). The development of the 15,000 SNP array and genotyping was performed by TraitGenetics GmbH (www.traitgenetics.com) and the SNPs represent a subset of markers from the 90,000 SNP array (Wang et al., 2014). The GBS data were generated and processed following established protocols (Himmelbach et al., 2014; Wendler et al., 2014). Briefly, digestion of genomic DNA was done with the enzymes PstI and MspI (New England Biolabs). Up to 190 individually barcoded samples were pooled per lane equimolarly and sequenced on the Illumina HiSeq 2000 device with 1 x 107 cycles in single-end mode using custom sequencing primer (Meyer and Kircher, 2010) according to the manufacturer's instructions. In total, five lanes of a single flow cell were sequenced with an average output of 3,052,589 raw reads per sample (ranging from 322,285 to 10,758,745 reads per sample) for 378 individuals (**Supplementary Table 1**). Following adapter trimming with cutadapt (Martin, 2011), reads were mapped to the reference genome sequence of bread wheat cultivar Chinese Spring (IWGSC, 2014) with BWA-MEM version 0.7.13 (r1126) (Li, 2013) using the -M option to mark shorter split hits as secondary. Mappings were transformed into the BAM format with SAMtools version 1.3 (Li et al, 2009). Novosort version 3.02.12 ^1^ was applied to sort and index records by position. BAM files were merged by genotype with Picard^2^. We called variants using the SAMtools/BCFtools pipeline version 1.3 (Li et al, 2009) with mpileup parameter set to “-DV”. A custom awk script was applied for initial filtering of genotype calls in the following manner: Bi-allelic sites with a minimum mapping quality score of 40 were called for homozygous and heterozygous genotype calls that were supported by at least two and four reads, respectively. We coded the SNP array and GBS marker data as (0, 1, 2, NA), where 0 and 2 represent the homozygous state for the first and second allele at a particular SNP locus, respectively, 1 represents the heterozygote class, and NA refers to missing values. As to multi-allelic SSR markers, if the allele appears for a certain genotype, it was coded as 1, if not, then 0. After that, this coding was also used for SSR markers assuming that each allele is a marker. We assessed the quality of the marker data in two steps: firstly, we deleted markers showing more than 5% of missing values, and then, we excluded the monomorphic markers [allele frequency (AF) = 0 or = 1]. After the quality assessment, 12,490 SNP array markers, 31,230 GBS markers, and 170 SSR alleles remained in the matrix. We then explored the genetic diversity based on these filtered markers without imputation and imputed the missing values according to the distribution of allele frequency for genomic prediction.

In order to compare properties between Elite and PGRs for each marker dataset, we calculated the minor allele frequency (MAF), population heterozygosity (H), and polymorphism information content (PIC). The standard deviations (SD) of these parameters were derived by means of bootstrapping with 1,000 rounds. We evaluated the genetic diversity from each group and calculated the Rogers' distances (RD) between pairs of genotypes. SDs were obtained by resampling genotypes without replacement with 1,000 rounds. Principal coordinates analysis (PCoA, Gower, 1966) was performed to investigate the population structure. PCoA was implemented with the function “cmdscale” from the R package “stats” ^3^. The relatedness of each pair of marker datasets was assessed through the Mantel correlation of their corresponding RD matrices (Mantel, 1967). Detailed information on the implementation of the population genetic analyses is outlined in the **Supplementary Material**.

### Field Trials and Phenotypic Data Analysis

For 339 genotypes (188 Elite and 151 PGR), phenotypic data were available. The 339 genotypes (or subsets) were phenotyped for grain yield (GY) (Mg ha^−1^) and heading date (HD) (days since 1 January) in three field experiments (**Table 1**). Experiment 1 comprised field trials of up to 278 genotypes evaluated in Gatersleben, Germany, and Malchow, Germany. The trials were performed in the year 2015 following an alpha-lattice design with two replicates (for details, see Philipp et al., 2018). Plot sizes were 5 m^2^ in Gatersleben and 3.75 m^2^ in Malchow. Experiment 2 included 166 out of the 188 elite lines and further 164 varieties (for details, see Zanke et al., 2014b; Kollers et al., 2013; Schulthess et al., 2017). Briefly, the experimental design was an alpha design with two replicates. The field trials were conducted in five locations during years 2009 and 2010, giving rise to eight location × year combinations (environments). Plot sizes ranged from 5 to 6.75 m^2^. Experiment 3 comprised field evaluation at five locations during 2016 and included 12 out of the 188 elite lines and 61 out of the 151 PGR. Briefly, the experimental design was an unreplicated alpha design (for details, see Boeven et al., 2019). Plot sizes ranged from 7.56 to 12 m². Across the three experiments, the 188 elite lines and the 151 PGR were evaluated in up to 15 environments for grain yield and in up to 11 environments for HD, respectively.

**Table 1:** Description of the environments used for evaluating grain yield and heading date (HD).

Experiment	Location	Year	No. of Elite	No. of PGR	Grain yield	Heading date (HD)
1	Gatersleben	2015	187	91	×	
	Malchow	2015	186 (184)*	91	×	×
2	Andelu	2009	166	0	×	×
	Andelu	2010	166	0	×	×
	Janville	2010	166	0	×	×
	Saultain	2010	166	0	×	×
	Seligenstadt	2009	166	0	×	×
	Seligenstadt	2010	166	0	×	×
	Wohlde	2009	166	0	×	×
	Wohlde	2010	166	0	×	×
3	Hohenheim	2016	12	61	×	×
	Renningen	2016	12	61	×	×
	Gatersleben	2016	12	61	×	
	Schackstedt	2016	12	61	×	
	Böhnshausen	2016	12	61	×	

* The number of elite lines for Malchow (2015) are different between grain yield (186) and HD (184).

We performed outlier tests and implemented a Bonferroni-Holm test standardized by the re-scaled median absolute deviation (MAD) (BH−MADR) at a significance level (P < 0.05) (Bernal-Vasquez et al., 2016). Thereafter, best linear unbiased estimations (BLUEs) and heritability for GY and HD were independently obtained using a two-stage approach.

First, BLUEs of each genotype within each single environment were estimated by fitting the following model:

(1) $$P\;\;=1_{n}\mu +G+R+B+e$$

in which, ***P*** contains the phenotypic values of GY or HD for each plot, ***µ*** corresponds to the overall mean, ***G*** represents the genotype effect, ***R*** stands for the effect of the replication, ***B*** is the effect of incomplete blocks, and ***e*** refers to the error term of the model. In the model, only ***µ*** and ***G*** were treated as fixed effect, while all other components were assumed to be random effects.

Second, the BLUEs of genotypes across all environments were estimated fitting the following model:

(2) $$Y=1_{n}\mu +G+E+G\times E+e$$

in which, ***Y*** contains the genotypic effects estimated within each environment using Equation (1), ***µ*** is the fixed effect of the overall mean, ***G*** corresponds to the fixed effects of genotypes across environments, ***E*** stands for random environment effects, *G*×*E* indicates the random effects of interaction between genotype and environment, and ***e*** is a random error term. Equations (1) and (2) were fitted using the mixed model R package ASReml-R (Butler et al., 2009).

Model (2) was also used to estimate the variances and heritability of each trait. During the computation for variances and heritability, ***µ*** is taken as fixed effect, while all other components in the model are assumed as random. Thereby, we calculated the broad-sense heritability (***H***^2^) as:

(3) $$H^{2}=\frac{\sigma_{G}^{2}}{\sigma_{G}^{2}+\sigma_{G\times E}^{2}/n+\sigma_{e}^{2}/\left(r\times \bar{n}\right)}$$

in which, $\sigma_{G}^{2}$ is the variance of genotypes, $\sigma_{G\times E}^{2}$ indicates the variance of genotype times environment interaction, $\sigma_{e}^{2}$ stands for the variance of error terms,$\;\bar{n}$ is the average number of environments in which genotypes were evaluated, and ***r*** represents the average number of replications.

### Genome-Wide Prediction

A genomic best linear unbiased prediction (GBLUP) model was implemented, with the co-variance matrix (G matrix) derived from SNP array, GBS, or SSR marker datasets. We employed single G matrix (single-kernel) or their combination (multi-kernel). The GBLUP model of the multi-kernel model was:

(4) $$Y=1_{n}\mu +g_{SNP}+g_{GBS}+g_{SSR}+e$$

Where ***Y*** contains the BLUEs for each trait, ***g***_***SNP***_,  ***g***_***GBS***_ and ***g***_***SSR***_ are random genetic effects derived from different markers, with $g_{SNP}∼N\left(0,\;A_{SNP}\sigma_{G_{1}}^{2}\right)$, $g_{GBS}∼N\left(0,A_{GBS}\sigma_{G_{2}}^{2}\right)$, $\;g_{SSR}∼N\left(0,A_{SSR}\sigma_{G_{3}}^{2}\right)$, and $e\;∼N\left(0,\text{I}\sigma_{e}^{2}\right)$, while ***A_SNP_***, ***A_GBS_*** and  ***A_SSR_*** are the numerator relationship matrix calculated using SNP array, GBS, or SSR marker datasets, respectively, according to VanRaden (2008) and $\sigma_{G_{1}}^{2}$ to $\sigma_{G_{3}}^{2}$ are the respective genetic variances of each component of the model. For single-kernel models, we used the ***g*_*SNP*_**, ***g*_*GBS*_**, and ***g*_*SSR*_** individually. The implementation of the models is described in detail in the **Supplementary Material**.

We applied a random resampling method for fivefold cross validation to investigate the prediction ability. In each cross validation, the population was divided into a training (80%) and a test set (20%). We used the training set to build the mixed model function, which was then used to predict the genetic value of the test set. The prediction ability was calculated as the Pearson correlation between estimated genetic values and the observed values in the test set. We performed 1,000 rounds of cross validation and recorded the mean and SD for these 1,000 correlation coefficients. The genomic prediction model was fitted using the “BGLR” R-package (Pérez and de los Campos, 2014). Besides GBS data generation, all computational methods were implemented in R environment (R 3.4.3, R Core Team, 2018).

## Results

### Molecular Diversity Estimated From SNP Array, GBS, and SSR Marker Data

We found for the SNP array markers ~5–6 times higher estimates of MAF, H, PIC, and RD than for the GBS markers considering the total population of 378 lines (**Table 2**; **Supplementary Figures 1** and **2**). In contrast, the values of H, PIC, and RD for the SNP array markers were only half as large as for the SSR markers, however, MAF for SNP array markers are roughly two times larger than for the SSR markers. Moreover, the mean values of these indices within the sample of 190 elite lines were generally lower compared to the population of PGR, regardless of the marker system. This shows the large molecular diversity of wheat accessions hosted at the genebank of the IPK Gatersleben.

**Table 2:** The mean and standard deviations (SD) of minor allele frequency (MAF), population heterozygosity (H), polymorphism information content (PIC), and average Rogers' distances (RD) for SNP array (SNP), genotyping-by-sequencing (GBS), and SSR markers.

Index	Marker set	All genotypes		Elite lines		PGRs (plant genetic resources)	
		Mean	SD	Mean	SD	Mean	SD
MAF	SNP	0.2438	0.0023	0.2172	0.0029	0.2480	0.0034
	GBS	0.0439	0.0006	0.0382	0.0005	0.0463	0.0009
	SSR	0.1382	0.0004	0.1381	0.0006	0.1385	0.0006
H	SNP	0.3299	0.0027	0.2961	0.0035	0.3336	0.0038
	GBS	0.0662	0.0009	0.0571	0.0008	0.0702	0.0015
	SSR	0.6765	0.0059	0.6286	0.0082	0.6924	0.0081
PIC	SNP	0.2418	0.0019	0.2177	0.0025	0.2443	0.0027
	GBS	0.0525	0.0008	0.0448	0.0006	0.0555	0.0012
	SSR	0.6449	0.0064	0.5930	0.0084	0.6655	0.0087
RD	SNP	0.3312	0.0528	0.2987	0.0472	0.3368	0.0532
	GBS	0.0651	0.0143	0.0561	0.0094	0.0696	0.0155
	SSR	0.6880	0.1190	0.6482	0.1186	0.7045	0.1190

**Figure 1:**
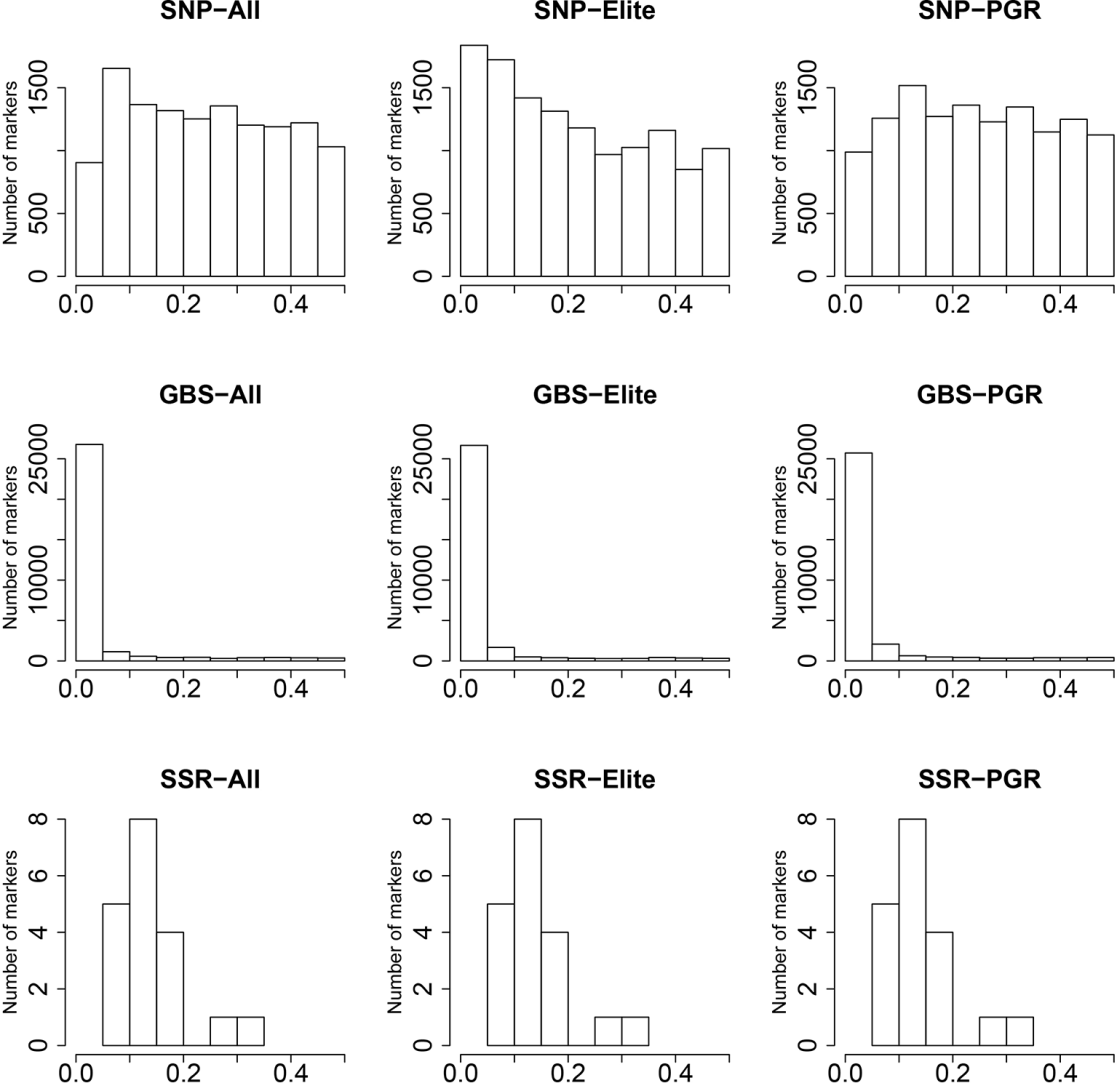
Distribution of minor allele frequencies (MAF) (x-axis) for single-nucleotide polymorphism (SNP) array, genotyping-by-sequencing (GBS), and SSR markers. Results are shown for the total population (All), the elite lines (Elite), and the plant genetic resources (PGR).

The SNP array markers followed a uniform pattern of MAF ranging from 0 to 0.5 (**Figure 1**), especially for the PGR population. In contrast, GBS markers were characterized by very low MAF in the range between 0 and 0.05. This suggests that GBS markers are more reliable in detecting the profile of rare alleles compared to SNP array markers. The distribution of MAF from SSR was derived from only 19 markers, and therefore the index spectra were quite sparse, which has to be considered when interpreting the results. In this context, we observed a peak at the MAF range between 0.05 and 0.2 for SSR markers.

The picture of the relatedness among the lines estimated on the basis of SNP array or GBS markers was similar (**Supplementary Figure 3**) and the correlation between distance matrices was up to r = 0.83 for the PGR population (**Table 3**). The correlations were significantly lower between SSR- and SNP array-based distance matrices with maximum r values of 0.48 and 0.52 when comparing SSR- with GBS-based and SNP array-based distance matrices, with both maximum values observed again in PGR.

**Table 3:** Correlation between Rogers’ distance (RD) matrixes calculated using data from SNP array (SNP), genotyping-by-sequencing (GBS), and SSR markers.

	All	Elite	PGR
SNP—GBS	0.818	0.683	0.830
GBS—SSR	0.454	0.414	0.476
SNP—SSR	0.500	0.442	0.520

Results are shown for the total population (All), the elite lines (Elite), and the plant genetic resources (PGR). Correlations were significantly (P < 0.001) larger than zero according to a Mantel test.

**Figure 2:**
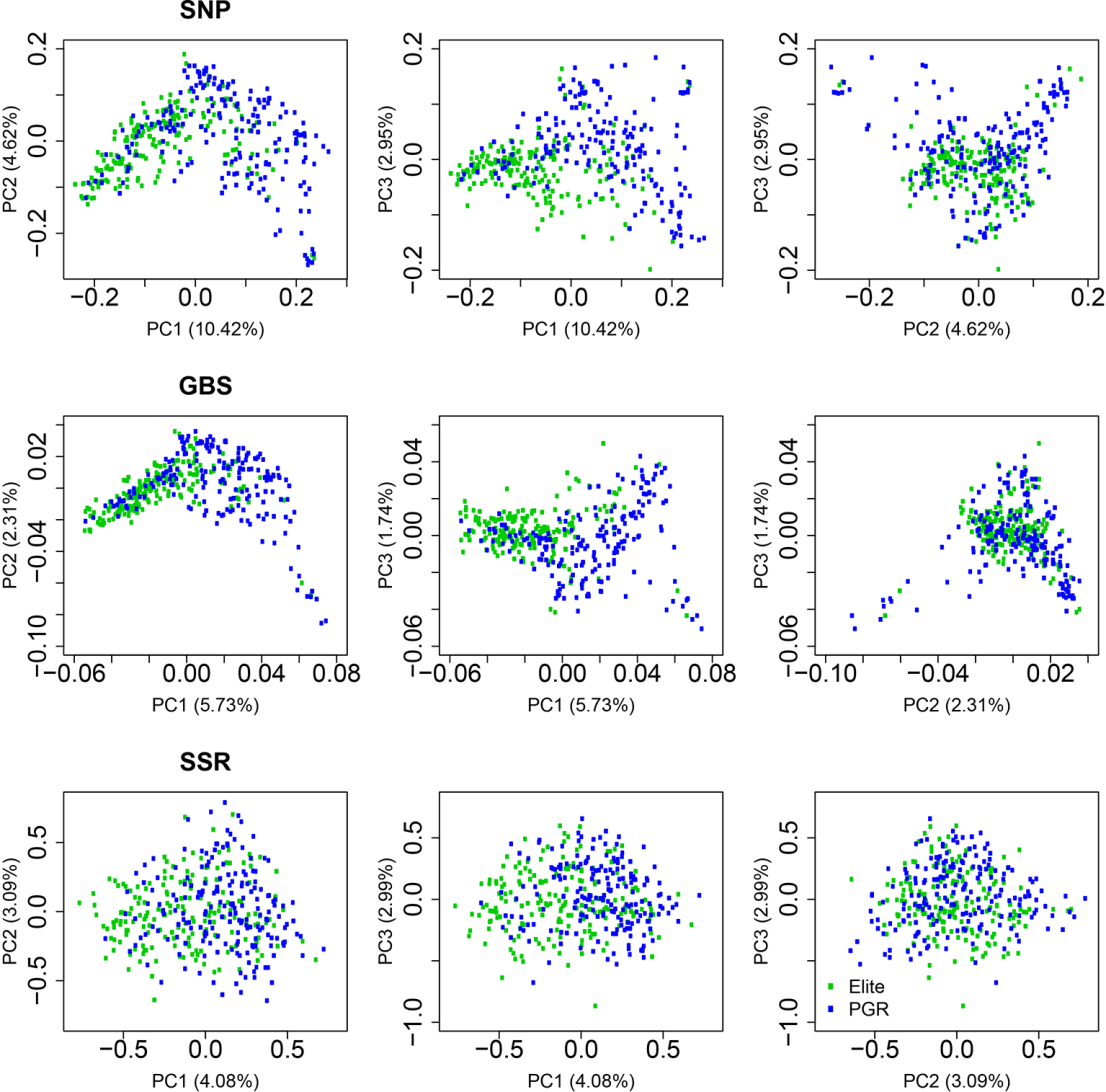
Principal coordinate analyses using data from single-nucleotide polymorphism (SNP) array, genotyping-by-sequencing (GBS), and SSR markers. Results are shown for the total population (All), the elite lines (Elite), and the plant genetic resources (PGR). PC1, PC2, and PC3 refer to the first, second, and third principal coordinate, respectively. Explained proportion of molecular variation is given in brackets.

The first, second, and third principal coordinates (PC1, PC2, and PC3) calculated based on the SNP-array data explained 10.42%, 4.62%, and 2.95% of the molecular variation, respectively (**Figure 2**, **Supplementary Table 2**). Elite lines and PGR were separated with respect to PC1. The distribution along PC2 and PC3 reflected the diversity within elite lines and PGR. A similar pattern was observed for the principle coordinate analysis based on the GBS data: Elite lines were separated from PGR with respect to PC1 and diversity within subpopulations was represented mainly by PC2 and PC3. The molecular variance explained by PC1, PC2, and PC3 was lower for the GBS compared to the SNP array data and amounted to 5.73%, 2.31%, and 1.74%, respectively. Similarly, the range of PC for the GBS marker was about 1/10 times of that of the SNP array data (**Figure 2**, **Supplementary Table 2**). For the SSR data, the differentiation between elite lines and PGR was less pronounced. In this case, PC1, PC2, and PC3 accounted for 4.08%, 3.09%, and 2.99% of the molecular variation.

### Comparison and Application of SNP Array, GBS, and SSR Markers in Genome-Wide Prediction

We estimated BLUEs of grain yield and HD for 339 of the 378 fingerprinted genotypes, including 188 Elite lines and 151 PGR. The BLUEs approached a bell-shaped distribution for both traits (**Supplementary Figure 4**). Heritability was 0.94 and 0.98 for grain yield and HD, respectively, which illustrates the high quality of the phenotypic data.

**Figure 3:**
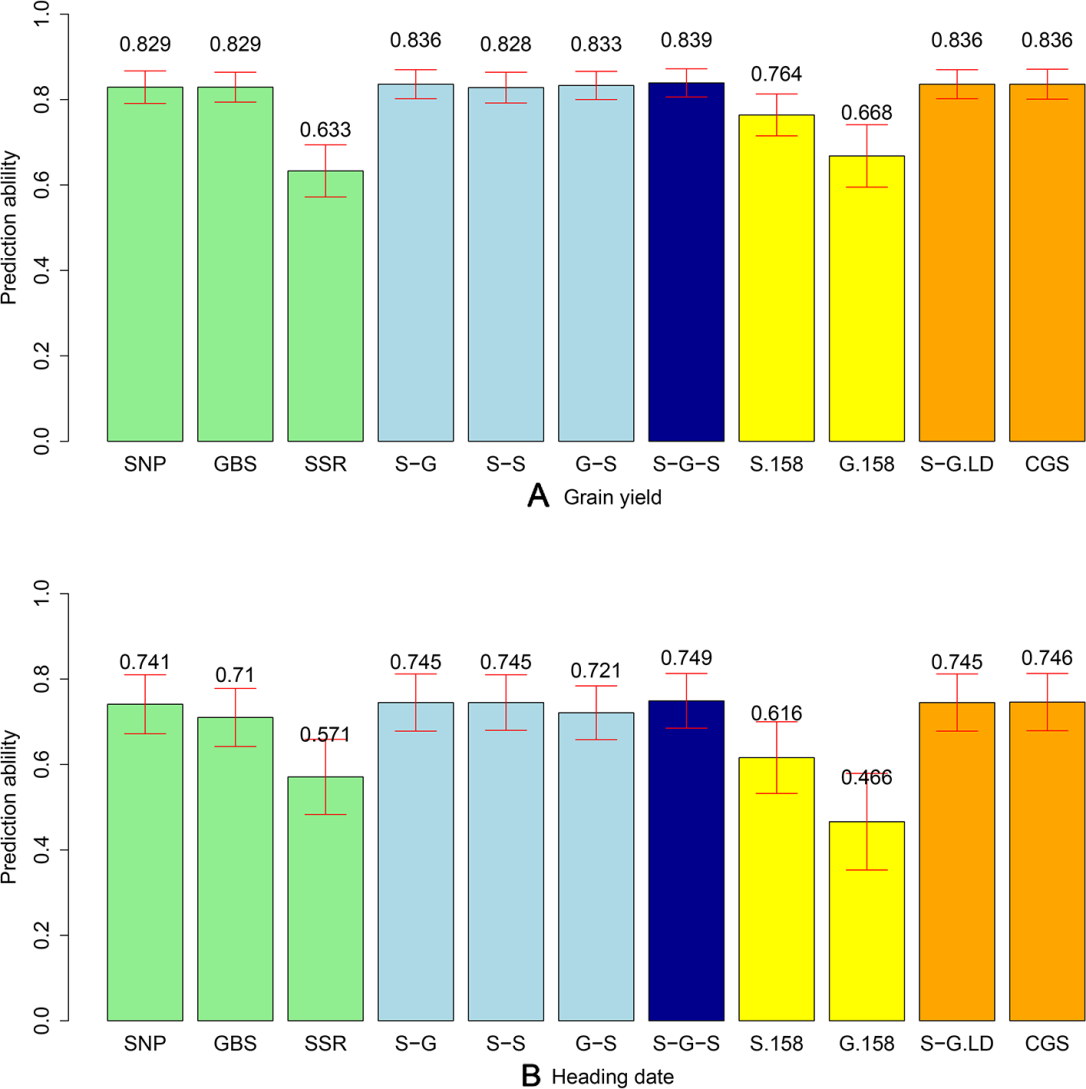
Bar plot of average prediction abilities derived from 1,000 cross-validations from different prediction models for **(A)** grain yield and **(B)** heading date (HD). Single kernel models (green) were used for data from single-nucleotide polymorphism (SNP) array, genotyping-by-sequencing (GBS), and SSR markers. Double-kernel models (light blue) were used combining SNP array and GBS markers (S-G), SNP array and SSR markers (S-S), as well as GBS and SSR markers (G-S). The three-kernel model (dark blue) combined SNP array, GBS, and SSR markers (S-G-S). Subsets of 158 markers from SNP array markers (S.158) and GBS markers (G.158) were used to run the single kernel models (yellow). Moreover, after ignoring the GBS markers with higher linkage equilibrium with SNP array markers, a double-kernel model combing SNP array and remained GBS markers (S-G.LD) and a single-kernel model of the combination of SNP array and remained GBS markers (CGS) (orange) were used. The corresponding standard deviations are illustrated as red bars.

The phenotypic data were combined with the different marker datasets and the prediction abilities for the combination of the different marker kernels in the total population of 339 lines were evaluated. We observed comparable prediction abilities for grain yield for the GBS and SNP array data, amounting to an average of 0.829 (**Figure 3**). The same picture was observed when comparing the prediction abilities for HD, but with a slightly lower level (0.741 and 0.710 for SNP array and GBS marker data, respectively). In contrast, the prediction abilities of SSR markers for grain yield (0.633) and HD (0.571) were significantly lower compared to SNP array and GBS markers. For grain yield, the prediction ability of the two-kernel model from the combination of SNP array and GBS markers (S-G) was slightly higher than that of the combination of GBS and SSR (G-S), followed by the combination of SNP array and SSR markers (S-S) (**Figure 3**). The highest prediction ability was achieved for the three-kernel model of the combination of SNP array, GBS, and SSR markers (S-G-S) (**Figure 3**). All in all, prediction abilities of the different kernel models were comparable with the only exception being the single model based on the G matrix derived from SSR markers. For the HD, the trends in prediction abilities of the different models were similar, but with lower values.

**Figure 4:**
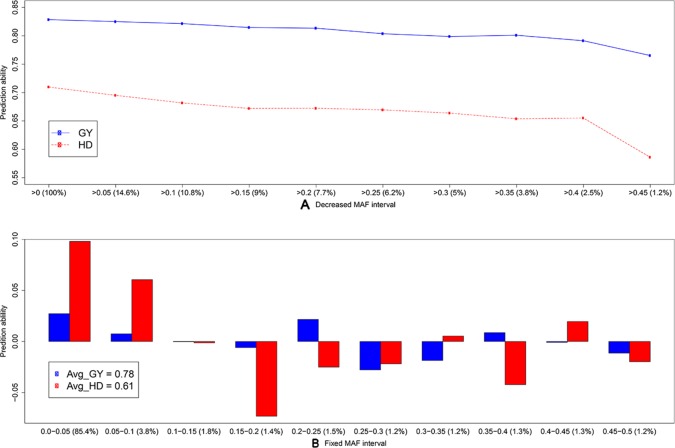
Average prediction ability derived from 1,000 cross-validation using the single-kernel model with a kernel matrix from genotyping-by-sequencing (GBS) markers for grain yield (GY, blue) and heading date (HD, red) for **(A)** decreased minor allele frequency (MAF) interval and **(B)** fixed MAF interval. The percentage (from the total number) of markers within frequency intervals is indicated within brackets. In **(B)**, bars indicate the average differences in prediction ability, and the average prediction abilities are indicated in the legend.

To discard the influence of marker density, we randomly selected 158 SNP array (S.158) or GBS markers (G.158), calculated the G matrices, and evaluated prediction abilities of single-kernel models applying cross validations. In general, the prediction ability of S.158 and G.158 was up to 34.4% lower than the total marker set (**Figure 3**). Interestingly, we observed lower prediction ability with the SSR compared to the S.158 and G.158 panels with the exception of the G.158 prediction for HD. In addition, the decrease in prediction ability was much more pronounced for the G.158 than for the S.158, suggesting an influence of the allele frequency distribution. We further inspected therefore the total set of GBS markers and tested the decrease in prediction abilities for GBS markers in dependence with MAF. The prediction ability decreased for both traits, grain yield and HD, with increasing thresholds of MAF (**Figure 4**). The number of markers decreased mostly in the interval between MAF 0 < 0.05. Thus, markers with very low MAF contributed substantially to the prediction ability for both traits, suggesting that they are actually important for genome-wide prediction.

Linkage disequilibrium (LD) between markers can impact the prediction ability for the multi-kernel models. We calculated therefore the LD between each pair of SNP array and GBS markers across the 339 lines and deleted the corresponding GBS markers if their LD was higher than r² = 0.95. After removing 2,826 (9.5%) GBS markers, which were in tight LD, we combined SNP array and remaining GBS markers to build a new dataset (CGS). We then did two *in-silico* experiments: first, we used the double-kernel model based on the SNP array and the GBS data excluding the linked markers (S.G.LD); second, we applied a single-kernel model for CGS. We observed for both traits that the performance of these two models was very close to that of S-G (**Figure 3**). Thus, the influence of linked markers was ignorable; however, if a huge number of markers are available, these results also indicate that the computational load can be decreased if linked markers are removed.

## Discussion

Data from GBS is typically characterized by a significant proportion of missing values (Elshire et al., 2011). We used a robust strategy to confront the challenges of dealing with missing values and, in a first step, filtered reliable SNPs with less than 5% of missing values. Then we imputed the missing values according to the original distribution of allele frequency for the implementation of genomic prediction. Nevertheless, it has already been shown that increasing the marker density beyond 3,000 SNPs in wheat populations of the size used in our study does not increase the genome-wide prediction ability nor does affect significantly the estimates of the relatedness among accessions (Liu et al., 2016). This is not the case for genome-wide association mapping studies, for which imputing missing values and increasing the marker density boosts the power of QTL detection (e.g., He et al., 2015; Negro et al., 2019). We would like to note that association mapping, however, was not the target of our study.

### Genotyping-By-Sequencing Enables Unbiased Estimates of the Genetic Diversity in Wheat Populations

Entire genebank collections have been fingerprinted using different marker technologies (e.g., Romay et al., 2013; Wang et al., 2018; Milner et al., 2019; Singh et al., 2019). In order to limit the costs, the sequence variation being represented is usually reduced. SSR markers, array-based scoring of SNPs, and GBS differ dramatically in the way sequence variation is reduced: GBS depends on the restriction enzymes used (Elshire et al., 2011), while SSR markers and also SNPs from arrays are selected using a subpopulation with limited size (Frascaroli et al., 2013). The 90k SNP array in wheat (Wang et al., 2014), for instance, was developed using data resulting from sequence information of 19 bread wheat and 18 tetraploid lines, as well as previous sequence information on 24 (M Ganal unpublished data; for details see Wang et al., 2014), 23 (Allen et al., 2011), 28 (Cavanagh et al., 2013), and 8 (Pont et al., 2013) wheat genotypes. The panel was selected to cover the global wheat diversity and included several elite wheat lines. The limited number of individuals used for SNP array discovery and the array design can led to a distorted picture of the molecular diversity denoted as ascertainment bias (Clark et al., 2005). Signs of an ascertainment bias are that rare alleles are missed, polymorphic markers have a high frequency of major alleles and genetic diversity is underestimated in the non-ascertained population (Clark et al., 2005). As already mentioned, H, PIC, and RD absolute estimates were ~5–6 times higher when computed from array-based SNPs than those obtained from GBS data (**Table 2**). Nevertheless, these results must be carefully interpreted, because this observation can be simply caused by a scale issue. In fact, we observed 23%, 24%, and 24% higher values based on H, PIC, and RD within the PGR population compared to elite lines as revealed by GBS, but this increased diversity amounted to only 13%, 12%, and 13% according to SNP array results, respectively. Moreover, for the SNP array data, the number of rare alleles was lower in the PGR population compared to elite lines (**Figure 1**). This was not the case for SNPs resulting from GBS data. Although it is true that the amount of SSR markers is substantially lower when compared to SNP array and GBS markers, which is mainly due to the high cost per data point of SSR markers, SSR markers are still being used by many researchers to study the genetic diversity existent in important crop species like potato (Wang et al., 2019), wheat (Sajjad et al, 2018), and maize (Adu et al, 2019). Moreover, it is interesting to observe that SSR markers are much capable to catch and portray the genetic diversity even with such a low number (19 markers and altogether 170 alleles). Altogether, these findings point to an underestimation of the diversity within the population of PGR versus the set of elite lines using the 90k SNP array, which can be explained by a large proportion of elite lines used to design the 90k SNP array.

The principal coordinate analyses revealed a comparable picture of the overall population structure across the three marker technologies (**Figure 2**). The total population clustered into a set of elite lines and PGRs. Similar findings have been reported by Cavanagh et al. (2013) investigating the diversity of 2,994 accessions of hexaploid wheat including landraces and modern cultivars and by Balfourier et al. (2019) examining the phylogeography of 4,506 landraces and cultivars originating from 105 different countries. Moreover, we observed that the estimates of the RD matrices using the array-based scoring of SNPs and GBS were similar, which is reflected by correlations for the total population of 0.83 (**Table 3**). This finding is in accordance with a previous study in wheat with U.S. elite lines (Elbasyoni et al., 2018) but also for other crops such as maize (e.g., Frascaroli et al., 2013) or barley genetic resources (Darrier et al., 2019). In contrast, the moderate correlations between distance matrices calculated based on SSR and GBS or SNP array markers (**Table 3**) are most likely caused by the limited number of SSR markers used in our study, which is in accordance with previous study in wild and cultivated barley (Hübner et al., 2012). This can be deduced from a high correlation (r = 0.85, P < 0.01) observed between kinship matrices calculated using a 90k SNP array and 782 SSR markers for 372 elite wheat lines observed in the study of Jiang et al. (2015). The low number of SSR markers, however, reflects comparable cost scenarios and shows that SSR markers are less suitable for large-scale characterization of wheat collections.

### Use of Genome-Wide Prediction to Provide Detailed Information for Entire Wheat Collections

More than half a million wheat genetic resources are conserved worldwide in genebanks (Longin and Reif, 2014). Detailed information on their phenotypic diversity is lacking, but is necessary to enable a targeted selection of promising accessions for (pre-)breeding. In a proof-of-concept study in sorghum, Yu et al. (2016) demonstrated the potential to use genome-wide predictions to efficiently provide phenotypic information about entire genebank collections. Our study confirmed the results in wheat for the two important agronomic traits grain yield and HD (**Figure 3**). The high prediction ability can be explained by the large genetic variation in our study. The population we used contained about 50% of PGRs, with grain yields ranging from 4.75 to 10.14 Mg ha^−1^ (**Supplementary Figure 4**) and a genetic variance of 0.98 (Mg ha^−1^)². We observed four times higher genetic variance compared to elite wheat lines in Europe (He et al., 2017). Although the genetic structure of the traits influences the prediction accuracy, it is difficult to say if this was the main driving factor of the prediction ability in our study. The lower predictability for HD reported in our study is consistent with the study of Bentley et al. (2014). They used a similar population size with 376 European elite wheat lines (from France, Germany, and the UK) and reported the average prediction accuracy of flowering time (0.52) to be considerably lower than grain yield (0.68), despite the higher heritability of flowering time compared to yield. The choice of marker systems did not strongly influence the prediction abilities, except for the SSR markers, which is presumably mainly due to the low number of markers (Jiang et al., 2014). Our results are consistent with a recent study in wheat that contrasted the potential and limitations of array-based scoring of SNPs and GBS to perform genome-wide prediction (Elbasyoni et al., 2018). The combination of marker information with two- or three-kernel models slightly improved prediction ability (**Figure 3**) and represents a solid approach for populations genotyped with different marker platforms. Interestingly, we found that very low frequency markers contributed to the improvement of prediction ability (**Figure 4**). However, such markers are usually deleted as outliers in SNP arrays but can be reliably captured by GBS. The potential of rare alleles to improve prediction ability combined with the expectation that valuable novel diversity is most likely rare (Mascher et al., 2019) suggests that rare markers deserve careful consideration in the design of the pre-breeding program.

## Conclusion

We observed an ascertainment bias for wheat caused by array-based SNP markers, which particularly impacts the estimates of the within population diversity. This was not the case with GBS, which makes it an interesting marker system to fingerprint entire genebank collections. In summary, our study showed the potential of genebank genomics to unlock the genetic diversity maintained in genebanks.

## Author’s Note

All authors declare that this study adheres to standard biosecurity and institutional safety procedures.

## Data Availability Statement

The datasets generated for this study are available on request to the corresponding author.

## Ethics Statement

All authors declare that this study adheres to ethical standards including ethics committee approval and consent procedure. All experiments were performed under the current laws of Germany.

## Author Contributions

JC, YZ, and JR designed the study. NS and MR contributed to the generation of genomic data. JC, SB, AS, and NP curated phenotypic and genomic data. JC performed the analyses. JC and JR wrote the paper with input from all co-authors.

## Conflict of Interest

The authors declare that the research was conducted in the absence of any commercial or financial relationships that could be construed as a potential conflict of interest.

## Footnotes

1 www.novocraft.com/documentation/novosort-2/

2 https://broadinstitute.github.io/picard/

3 https://cran.r-project.org/web/packages/STAT/STAT.pdf
