# Supplementary material for: Suitability of Single-Nucleotide Polymorphism Arrays Versus Genotyping-By-Sequencing for Genebank Genomics in Wheat
Source: Front Plant Sci. 2020 Feb 14;11:42. doi: 10.3389/fpls.2020.00042 (PMC7033508; doi:10.3389/fpls.2020.00042)
Supplement: Supplementary file 1 [file DataSheet_1.pdf]

Supplementary Material

1 Supplementary methods and models

For both SNP array and GBS markers, the calculation is based on each locus, but for SSR markers, every allele from every marker is considered as a locus.

1.1 Minor Allele Frequency (MAF)

For SNP array and GBS datasets, the marker values were coded as 0, 1, 2 and NA. Therefore, if there are n genotypes across one locus, then,

$$\boldsymbol{n = n_2 + n_1 + n_0} \tag{1}$$

in which, n_2, n_1, and n_0 are the amounts for the values "2", "1", and "0", respectively.

The allele frequency (AF) and the MAF for the i-*th* marker is:

$$\boldsymbol{AF_i = \frac{2\times n_2+1\times n_1+0\times n_0}{2n} = \frac{(\sum_{k=1}^{n} g_k)/n}{2} = \frac{\bar{g}_i}{2}} \tag{2}$$

and

$$\boldsymbol{MAF_i = min\big(AF_i, (1 - AF_i)\big).} \tag{3}$$

in which, k is the kth genotype, and $g_k$refers to the marker value of the kth genotype.

For each SSR allele, the allele frequency is in fact the occurring frequency, which is always not higher than 0.5, thereby,

$$\boldsymbol{MAF_i = AF_i = \ \bar{g}_i.} \tag{4}$$

We used the average of MAF_i from one SSR marker as the MAF of that marker.

1.2 Population Heterozygosity (H)

Once the allele frequency of each allele is determined, H will be retrieved to measure the gene-frequency variation. H is applicable in any population regardless of the number of alleles at a locus or the pattern of evolutionary forces, as well as in any organism without considering its reproduction (breeding) way or chromosome ploidy (Nei, 1973). H is calculated as the following (Nei, 1973, Nagy et al., 2012):

$$\boldsymbol{H = 1 - \sum_{i=1}^{l} AF_i^2} \tag{5}$$

where $AF_i$is the frequency of the ith allele and $\boldsymbol{l}$ corresponds to the total number of alleles.

The biallelic nature ($AF_1 + AF_2 = 1$) of SNPs markers (SNP array and GBS datasets), reduces H to:

$$H = 1 - \sum_{i=1}^{2} AF_i^2 = 1 - \left(AF_1^2 + AF_2^2\right) = 2AF_1 \times AF_2 \,. \tag{6}$$

1.3 Polymorphism Information Content (PIC)

For each marker locus, PIC is defined as (Botstein et al., 1980, Nagy et al., 2012):

$$PIC = 1 - \sum_{i=1}^{l} AF_i^2 - \sum_{i=1}^{l-1} \sum_{j=i+1}^{l} 2\, AF_i^2 \times AF_j^2$$

$$= H - \sum_{i=1}^{l-1} \sum_{j=i+1}^{l} 2\, AF_i^2 \times AF_j^2. \tag{7}$$

PIC is thereby considered as the corrected heterozygosity with the information from partially mating (Hildebrand et al., 1992).

For both, SNP array and GBS datasets:

$$PIC = 2AF_1 \times AF_2 - 2AF_1^2 \times AF_2^2 = H - 2AF_1^2 \times AF_2^2 \tag{8}$$

The maximum PIC for biallelic markers is 0.375. Markers having more alleles will be more informative, and their corresponding PIC is higher.

1.4 Rogers' Distance (RD)

The Rogers' Distance (RD) is used as an index for measuring the genetic distance between two genotypes according to the alleles within each marker system as:

$$RD = \frac{1}{m} \sum_{i=1}^{m} \sqrt{\frac{1}{2} \sum_{j=1}^{n_j} \left(p_{ij} - q_{ij}\right)^2}, \tag{9}$$

with m referring to the number of loci, n_i being the number of alleles at the ith loci, while p_{ij} and q_{ij} correspond to the allele frequencies of the jth allele at the ith locus from two genotypes. RD is a measurement of gene diversity. Moreover, we estimated also the genetic similarity as 1 - RD.

1.5 Single-Kernel model

The general form of the single kernel model is the following:

$$Y = 1_n \mu + Z_A \alpha + e \tag{10}$$

in which, μ is the overall mean, α corresponds to additive effects, n denotes the number of lines, p indicates the number of markers, Z_A represents the design matrix with the dimension of $(n \times p)$ for the additive effects of the marker SNP array, GBS, SSR, individually, and 1_n is the vector of length n containing only ones.

The additive model (10) is equivalent to a GBLUP model:

$$Y = 1_n \mu + g + e \tag{11}$$

where g $\sim N(0, A * \sigma_G^2)$, and e $\sim N(0, \sigma_e^2)$, and A is the numerator relationship matrix, and is calculated according to VanRaden (2008). This A matrix will be used as kernel matrix (K) for the single kernel method in the R BGLR-Package.

1.6 Multi-Kernel model

In order to test whether the different marker system can be complementary to each other, we also used a model combining two or three marker data together. In general, the multi-kernel model uses all of three marker data:

$$\boldsymbol{Y} = \boldsymbol{1_n \mu} + \boldsymbol{Z_{SNP} \alpha} + \boldsymbol{Z_{GBS} \beta} + \boldsymbol{Z_{SSR} \gamma} + \boldsymbol{e} \quad (12)$$

Here α, β and γ are additive effects for SNP array, GBS, and SSR marker, respectively. Z_{SNP}, Z_{GBS} and Z_{SSR} are the design matrix with the dimension of $(n \times p)$ for the related additive effects of each marker data. The row dimension of Z_{SNP}, Z_{GBS} and Z_{SSR} is n, and n is number of genotypes. The column dimensions of them are the number of markers (p) for each type of marker data.

The equivalent GBLUP model of multi-kernel model (11) will be:

$$\boldsymbol{Y} = \boldsymbol{1_n \mu} + \boldsymbol{g_{SNP}} + \boldsymbol{g_{GBS}} + \boldsymbol{g_{SSR}} + \boldsymbol{e} \quad (13)$$

While $g_{SNP} \sim N(0, A_{SNP} \sigma_{G_1}^2)$, $g_{GBS} \sim N(0, A_{GBS} \sigma_{G_2}^2)$, $g_{SSR} \sim N(0, A_{SSR} \sigma_{G_3}^2)$,and e $\sim N(0, I\sigma_e^2)$, and $A_{SNP}, A_{GBS}, A_{SSR}$ are the numerator relationship matrix also calculated according to VanRaden (2008). These numerator relationship matrices will be used as kernel matrix for the multi-kernel methods in the R BGLR-package.

We also tested all three possible combinations of two marker data: SNP array plus GBS markers, SNP array plus SSR markers, and SSR plus GBS markers. For this purpose, we just needed to remove the unused marker effect from the model (13).

1.7 Linkage disequilibrium

Linkage disequilibrium (LD) in the form of the squared Pearson coefficient of correlation (r2) was used as a measure of non-random association between two different loci (Hill and Robertson, 1968).

2 Supplementary Tables and Figures

Supplementary Table 1. The raw reads per sample during the sequencing of GBS markers.

No.	Genotype Name	Reads	No.	Genotype Name	Reads
1	Achat	3,621,856	190	TRI11372	2,649,516
2	Adler	2,456,605	191	TRI11374	7,622,673
3	Alceste	3,578,069	192	TRI11439	2,092,928
4	Alchemy	1,644,484	193	TRI11469	4,590,339
5	Ambition	2,560,500	194	TRI11767	3,231,833
6	Ambrosia	8,044,290	195	TRI11784	1,782,905
7	Amundsen	2,527,594	196	TRI11826	1,929,382
8	Andi	1,764,812	197	TRI119	1,546,514
9	Antonius	1,066,850	198	TRI12010	2,588,285
10	Aperitiv	2,360,246	199	TRI12072	3,081,806
11	Apollo	1,498,365	200	TRI1218	2,727,415
12	Ares	2,478,162	201	TRI12313	3,669,136
13	Arina	1,748,463	202	TRI12594	2,177,919
14	B19	5,463,066	203	TRI12707	4,291,796
15	B47	2,585,870	204	TRI1273	4,660,388
16	B52	3,167,027	205	TRI12829	3,103,135
17	B87	2,706,501	206	TRI13098	3,112,168
18	B98	4,330,448	207	TRI13141	2,921,678
19	Baguette11	3,996,399	208	TRI13212	2,166,360
20	Banderola	2,458,957	209	TRI13344	5,024,556
21	Banquet	5,581,020	210	TRI1340	3,994,957
22	Battalion	2,012,527	211	TRI13430	2,855,090
23	Batuta	4,407,480	212	TRI13672	2,653,439
24	Bill	2,101,068	213	TRI1388	1,657,390
25	Bogatka	2,106,821	214	TRI14052	3,772,499
26	Boregar	3,368,843	215	TRI14068	1,592,321
27	Brompton	2,070,234	216	TRI14280	1,899,906
28	Bryza	3,961,502	217	TRI14318	3,092,516
29	Caesar	1,540,384	218	TRI14868	2,763,915
30	Capo	1,487,832	219	TRI14884	3,984,938
31	Cassiopeia	3,257,032	220	TRI14887	2,014,369
32	Consort	4,579,806	221	TRI14895	2,775,942
33	Contur	4,541,109	222	TRI14905	2,222,273
34	Cordiale	2,784,914	223	TRI14934	3,518,964
35	Corsaire	1,198,837	224	TRI14997	1,638,323
36	CPBTW130	3,718,315	225	TRI15098	3,181,676
37	Deben	1,792,766	226	TRI1571	2,435,964
38	Duxford	1,762,599	227	TRI1591	3,473,771
39	Einstein	2,420,913	228	TRI16	3,277,508

40	Eriwan	4,688,976	229	TRI1601	3,380,321
41	Estica	4,685,454	230	TRI16546	3,396,113
42	Eurojet	2,296,198	231	TRI16553	1,503,646
43	Expert	2,780,237	232	TRI16644	3,336,969
44	Exsept	2,415,202	233	TRI16696	2,955,098
45	Fastnet	2,469,749	234	TRI16698	2,880,213
46	Fidelius	2,859,296	235	TRI16767	3,551,402
47	Figura	4,053,400	236	TRI16818	3,287,540
48	Finezja	3,527,465	237	TRI16935	5,096,841
49	Fregata	2,544,266	238	TRI16948	1,917,757
50	Fridolin	2,234,913	239	TRI17248	4,064,615
51	Frument	7,255,066	240	TRI19517	3,735,811
52	Gallant	3,568,801	241	TRI19607	1,838,600
53	Gatsby	5,429,109	242	TRI19890	2,023,497
54	Gladiator	3,154,784	243	TRI21034	2,640,862
55	Glasgow	4,682,545	244	TRI21059	4,632,390
56	Glaucus	3,521,772	245	TRI21088	2,174,491
57	Globus	2,153,738	246	TRI21165	2,063,041
58	Gulliver	1,897,292	247	TRI21173	2,660,532
59	H05606	4,383,065	248	TRI21181	1,829,014
60	Hanseat	2,571,823	249	TRI21330	1,486,907
61	Haven	3,942,631	250	TRI21379	4,756,600
62	Hereford	3,914,920	251	TRI21447	3,317,361
63	Hereward	2,872,166	252	TRI21847	1,392,242
64	Herzog	1,260,288	253	TRI21888	1,872,073
65	Humber	1,238,478	254	TRI22325	4,129,777
66	Hussar	2,840,966	255	TRI22800	2,004,659
67	Hyperion	3,246,259	256	TRI2287	2,587,022
68	Ibis	1,616,597	257	TRI23047	3,987,360
69	Instinct	1,937,267	258	TRI23126	2,890,851
70	Intact	3,114,204	259	TRI23266	2,421,319
71	Intense	2,956,576	260	TRI23279	3,574,510
72	Isengrain	1,425,552	261	TRI2342	2,274,206
73	Istabraq	4,007,347	262	TRI2353	3,006,312
74	Izyda	1,527,813	263	TRI23671	3,282,643
75	Jantarka	3,606,674	264	TRI23739	1,197,241
76	Kampana	2,678,655	265	TRI23767	3,173,234
77	Kanzler	2,183,069	266	TRI23983	4,741,564
78	Ketchum	3,359,826	267	TRI24049	3,108,512
79	Kingdom	2,353,094	268	TRI24071	3,183,872
80	Kleber	2,185,349	269	TRI24096	2,540,372
81	Kobiera	2,236,380	270	TRI2411	4,214,298
82	KobraPlus	2,983,658	271	TRI2422	4,532,510
83	Kohelia	2,506,917	272	TRI24232	2,900,086

84	Korweta	6,667,730	273	TRI243	3,935,071
85	Kosack	4,785,684	274	TRI24352	4,255,518
86	KWSAurumLP819404	2,100,241	275	TRI24403	1,175,174
87	KWSChamsinLP779	3,123,669	276	TRI24519	2,287,543
88	KWSErasmus	4,451,781	277	TRI24652	3,014,640
89	KWSOzon	2,328,551	278	TRI24730	4,536,400
90	KWSSciroccoLP509304	3,092,327	279	TRI24731	1,730,355
91	Lars	3,039,392	280	TRI25204	2,304,875
92	Legenda	4,878,642	281	TRI25248	2,400,336
93	Limerick	3,594,101	282	TRI25292	3,326,110
94	Lona	1,500,409	283	TRI25327	2,749,638
95	Lynx	2,587,205	284	TRI25909	3,173,461
96	Macro	4,791,086	285	TRI28909	4,336,216
97	Malacca	6,851,497	286	TRI29458	2,188,799
98	Marin	1,484,124	287	TRI33	5,100,791
99	Markiza	2,921,687	288	TRI3372	2,635,885
100	Marksman	1,495,472	289	TRI3373	3,852,211
101	Mascot	3,098,510	290	TRI344	3,623,538
102	Mewa	2,291,694	291	TRI355	4,500,720
103	Mikon	3,201,468	292	TRI36	3,495,381
104	Monsun	2,075,043	293	TRI370	3,477,963
105	Monty	1,484,069	294	TRI3788	2,124,835
106	Muskat	3,858,160	295	TRI3792	1,693,348
107	Musketeer	4,200,598	296	TRI3818	4,809,274
108	Muszelka	1,389,031	297	TRI4	2,573,278
109	Muza	7,045,164	298	TRI4249	3,255,641
110	Nadobna	1,192,420	299	TRI4256	2,317,926
111	Naridana	4,721,997	300	TRI4526	1,880,680
112	Nateja	2,637,684	301	TRI4589	3,667,734
113	Natula	5,445,339	302	TRI4639	4,766,414
114	Nutka	2,227,445	303	TRI4742	2,816,979
115	Oakley	3,915,843	304	TRI4765	2,307,598
116	Orestis	2,857,585	305	TRI4808	2,021,226
117	OstkaStrzelecka	4,929,945	306	TRI4840	2,439,214
118	Ostroga	2,113,469	307	TRI4884	4,622,635
119	P05311	322,285	308	TRI4955	4,609,460
120	P05312	3,653,197	309	TRI4957	3,203,753
121	P06079	3,222,518	310	TRI4968	3,695,629
122	Philius	5,027,613	311	TRI4980	2,892,785
123	Portland	2,037,364	312	TRI5081	3,538,266
124	Position	4,049,529	313	TRI5082	3,702,445
125	Potenzial	2,699,885	314	TRI5085	5,051,487
126	Prinz	2,629,221	315	TRI5121	1,795,573

127	Rainer	2,432,251	316	TRI5153	2,293,675
128	Ramiro	2,026,692	317	TRI5164	2,447,613
129	Renan	2,692,876	318	TRI5167	2,501,208
130	Rialto	3,527,482	319	TRI5180	3,333,282
131	Robigus	3,346,228	320	TRI52	1,240,751
132	Rubens	4,834,977	321	TRI5400	2,690,182
133	Rywalka	3,960,823	322	TRI56	1,424,481
134	Sailor	1,353,444	323	TRI58	1,854,431
135	Sakura	3,183,102	324	TRI63	2,007,843
136	Satyna	4,247,533	325	TRI6729	3,319,329
137	Savannah	2,996,141	326	TRI6787	2,233,448
138	Semper	2,709,966	327	TRI6811	1,479,513
139	Senat	1,654,169	328	TRI6816	3,799,456
140	Skalmeje	4,519,010	329	TRI6838	2,373,654
141	Smuga	3,540,325	330	TRI6840	1,350,186
142	Smuggler	3,883,807	331	TRI6876	3,389,997
143	Sobi	3,369,595	332	TRI6899	2,882,215
144	Soissons	2,487,493	333	TRI7028	2,986,961
145	Sokrates	4,266,854	334	TRI7399	2,628,868
146	Solist	3,998,516	335	TRI75	2,766,893
147	Solitaer	3,244,292	336	TRI7709	4,055,209
148	Solstice	3,826,632	337	TRI7838	3,216,067
149	Sophytra	2,813,906	338	TRI7893	4,855,432
150	Sperber	4,658,344	339	TRI7918	1,907,717
151	Stava	2,686,837	340	TRI8	3,496,604
152	Striker	2,679,345	341	TRI8030	2,181,813
153	Sukces	1,424,725	342	TRI8097	2,393,806
154	Sultan	2,595,681	343	TRI8130	2,969,458
155	SWHarnesk	3,664,215	344	TRI8134	2,594,365
156	SWHarpun	1,485,849	345	TRI8137	3,263,341
157	SWHurtig	3,470,890	346	TRI8280	1,695,400
158	SWSkotte	3,982,545	347	TRI8301	1,845,074
159	SWTataros	4,178,868	348	TRI84	1,615,703
160	SWTopper	2,517,664	349	TRI8402	1,386,462
161	Symbol	3,801,217	350	TRI93	2,402,380
162	Taifun	3,216,003	351	TRI9332	4,473,896
163	Tambor	2,736,812	352	TRI9366	2,415,614
164	Tarkus	1,722,523	353	TRI9389	5,368,655
165	Tarso	3,009,408	354	TRI9397	2,826,564
166	Timber	2,391,875	355	TRI9410	1,675,162
167	Tommi	1,792,725	356	TRI9433	3,581,090
168	Tonacja	5,318,414	357	TRI9670	2,203,954
169	Toras	2,285,671	358	TRI9968	5,523,978
170	Torkil	3,766,767	359	TRI9993	1,831,002

171	Toronto	2,693,228	360	Trintella	2,457,146
172	Torrild	4,604,507	361	Tuareg	3,407,801
173	Tower	4,509,378	362	Tuerkis	2,146,844
174	Transit	3,826,600	363	Tukan	4,445,462
175	Trappe	2,616,527	364	Tulsa	2,511,746
176	Travix	1,657,491	365	Tuscan	3,414,422
177	TRI10149	3,882,685	366	Vanek	3,481,589
178	TRI10316	1,083,530	367	Velocity	3,646,481
179	TRI10384	2,524,376	368	ViscountCPBTW136	3,797,464
180	TRI10401	3,462,662	369	Vitus	2,517,378
181	TRI1068	2,032,574	370	Vivant	4,270,094
182	TRI1081	3,078,915	371	Welford	3,916,213
183	TRI10924	2,349,698	372	Winnetou	4,171,168
184	TRI10952	1,609,912	373	Wydma	2,478,829
185	TRI11057	1,936,425	374	Xenos	2,321,703
186	TRI1109	10,758,745	375	Xi19	1,929,533
187	TRI11226	1,754,750	376	Zanatan	1,822,008
188	TRI11301	4,899,017	377	Zawisza	1,818,627
189	TRI1135	3,514,249	378	Zebedee	2,294,901

Supplementary Table 2. The explained percentage of molecular variation of the first 10 principal coordinates derived for SNP array (SNP), genotyping-by-sequencing (GBS) and SSR markers. Results are shown for the total population (All), the elite lines (Elite), and the plant genetic resources (PGR).

Principal Coordinate (%)	All			Elite			PGR		
	SNP	GBS	SSR	SNP	GBS	SSR	SNP	GBS	SSR
1	10.42	5.73	4.08	9.90	5.91	5.52	11.43	9.19	6.23
2	4.62	2.31	3.09	5.14	3.22	4.41	6.57	5.23	4.20
3	2.95	1.74	2.99	3.60	3.06	3.55	4.40	3.73	3.49
4	2.19	1.42	2.29	3.24	2.24	3.31	2.90	2.88	3.45
5	2.10	1.37	2.11	2.69	1.92	3.18	2.28	2.61	3.06
6	1.81	1.20	2.04	2.67	1.87	2.94	2.21	2.01	2.89
7	1.71	1.10	1.97	2.49	1.74	2.80	1.79	1.84	2.69
8	1.57	0.99	1.81	2.11	1.49	2.54	1.74	1.64	2.49
9	1.35	0.92	1.72	1.92	1.43	2.41	1.70	1.59	2.38
10	1.23	0.83	1.71	1.85	1.40	2.22	1.58	1.52	2.26
Sum	29.94	17.62	23.82	35.61	24.27	32.88	36.62	32.23	33.15

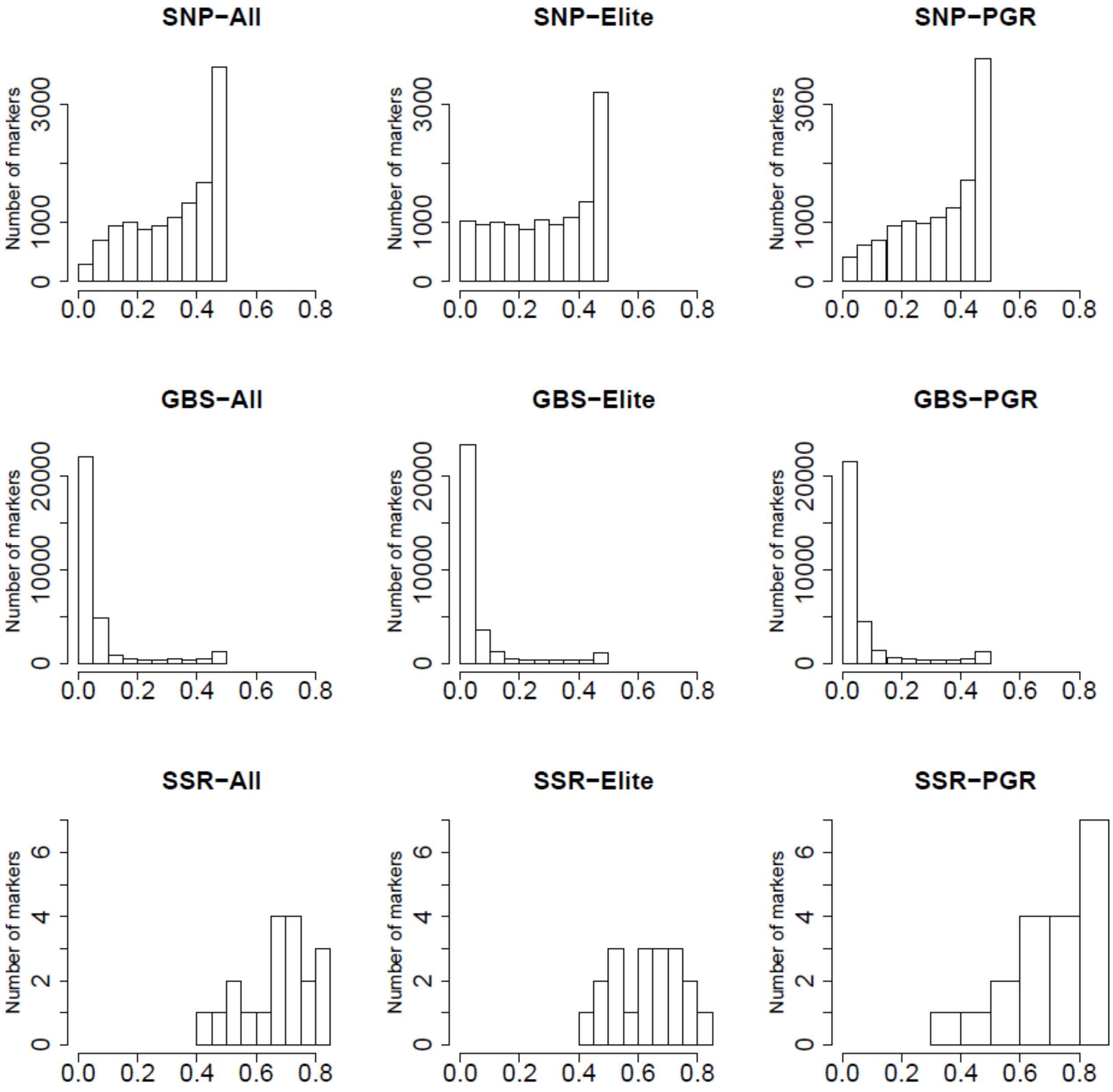

Supplementary Figure 1. Distributions of population heterozygosity (x-axis) for SNP array (SNP), genotyping-by-sequencing (GBS) and SSR markers. Results are shown for the total population (All), the elite lines (Elite), and the plant genetic resources (PGR).

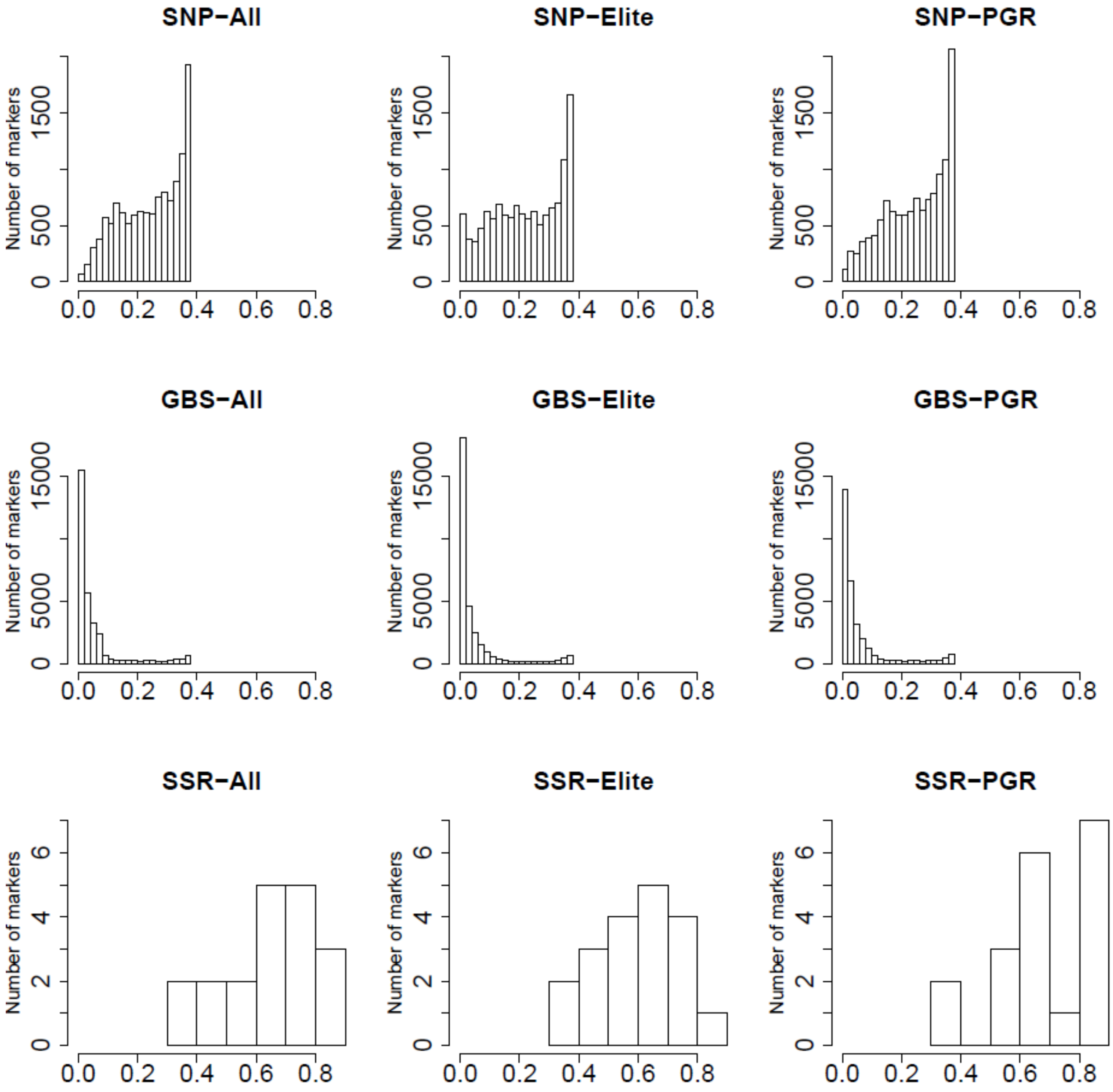

Supplementary Figure 2. Distributions of polymorphism information content (PIC) (x-axis) for SNP array (SNP), genotyping-by-sequencing (GBS) and SSR markers. Results are shown for the total population (All), the elite lines (Elite), and the plant genetic resources (PGR).

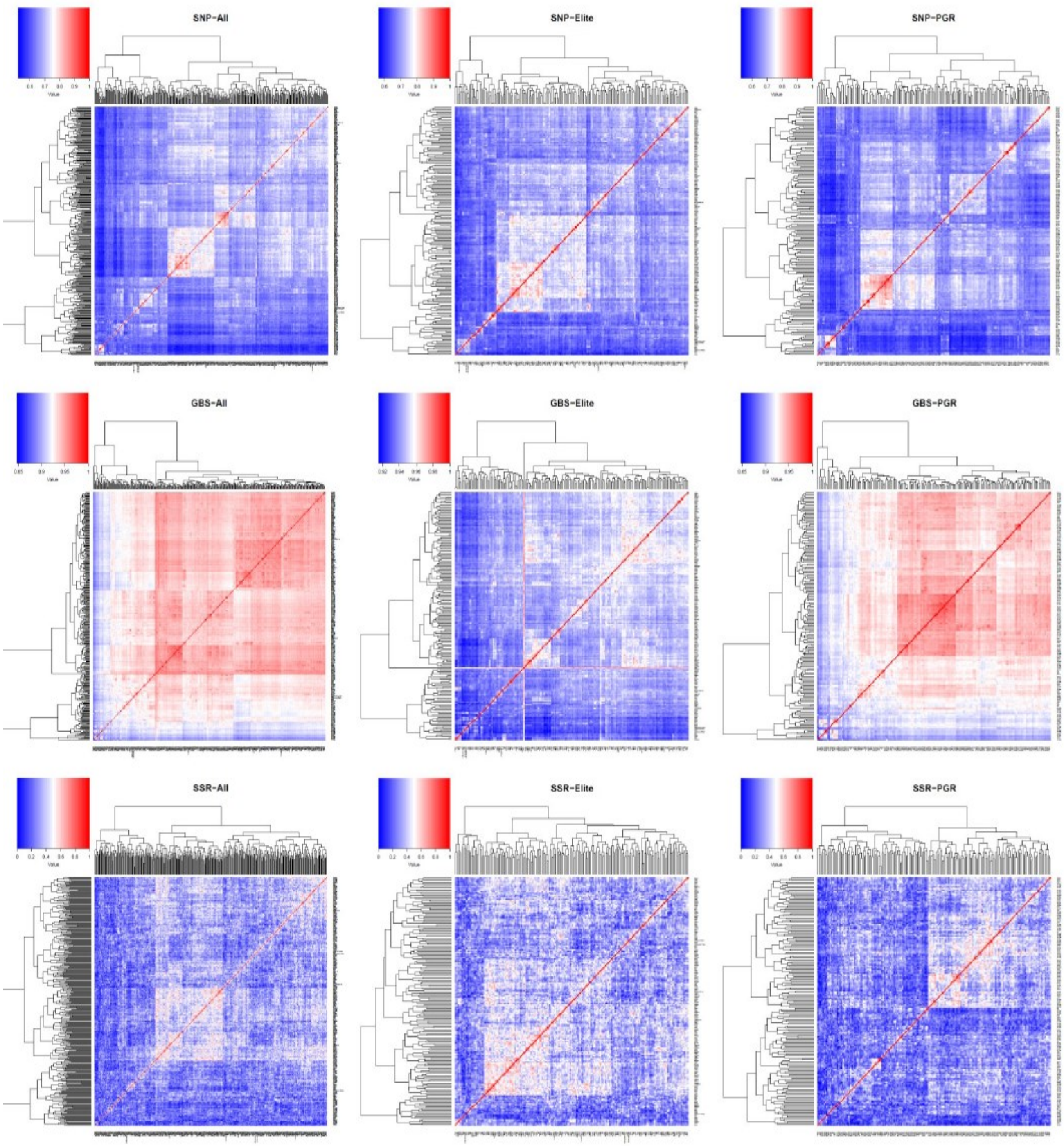

Supplementary Figure 3. Heat map plots of genetic similarity (1 - Rogers' distances) estimated based on SNP array (SNP), genotyping-by-sequencing (GBS) and SSR markers. Results are shown for the total population (All), the elite lines (Elite), and the plant genetic resources (PGR).

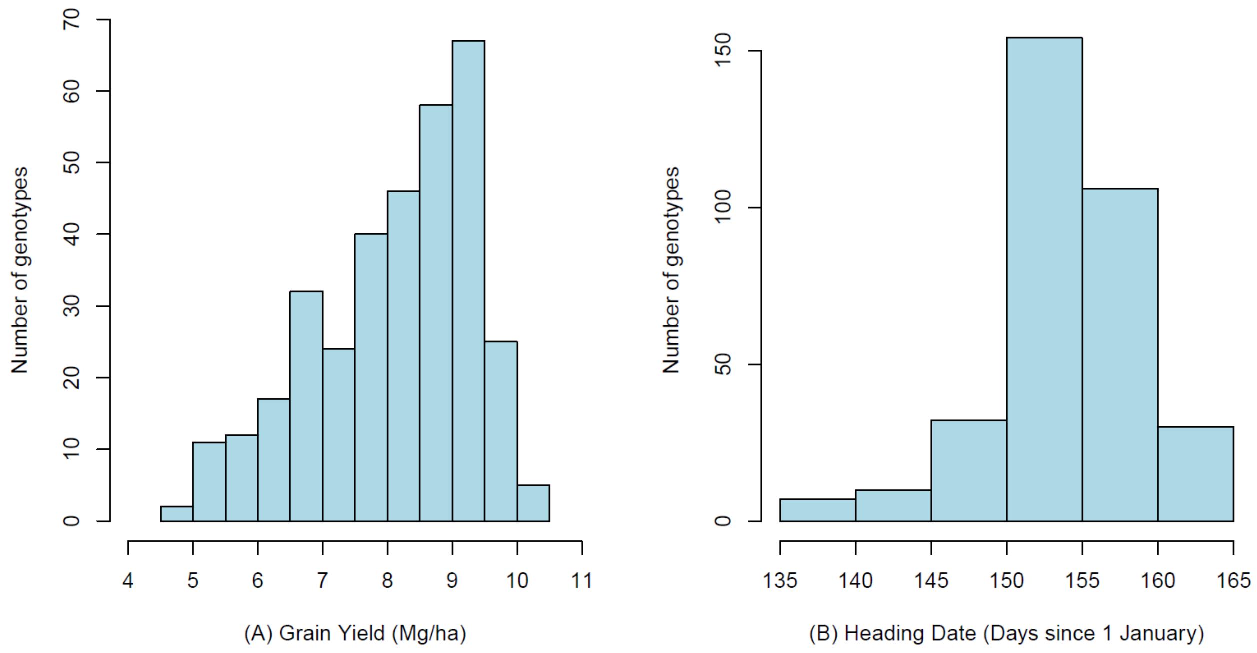

Supplementary Figure 4. Histograms of best linear unbiased estimations for (A) Grain yield (Mg/ha) and (B) Heading date (days since 1 January) for 339 wheat genotypes.

Reference

Botstein, D., White, R.L., Skolnick, M. and Davis, R.W. (1980). Construction of a genetic linkage map in man using restriction fragment length polymorphisms. *Am J Hum Genet* 32, 314-331.Hildebrand, C.E., Torney, D.C. and Wagner R.P. (1992). Informativeness of polymorphic DNA markers. *Los Alamos Science* 20, 100-102.

Hildebrand, C.E., Torney, D.C. and Wagner, R.P. (1992). Informativeness of polymorphic DNA markers. *Los Alamos Sci.* 20, 100.102.

Hill, W.G. and Robertson, A. (1968). Linkage disequilibrium in finite populations. *Theor. Appl. Genet.* 38, 226-231. doi: 10.1007/BF01245622

Nagy, S., Poczai, P., Cernak, I., Gorji, A.M., Hegedus, G. and Taller, J. (2012). PICcalc: An online program to calculate polymorphic information content for molecular genetic studies. *Biochem. Genet.* 50, 670-672. doi: 10.1007/s10528-012-9509-1

Nei, M. (1973). Analysis of gene diversity in subdivided populations. *Proc. Natl. Acad. Sci. U.S.A.* 70 (12), 3321-3323. doi: 10.1073/pnas.70.12.3321

VanRaden, P.M. (2008). Efficient methods to compute genomic predictions. *J. Dairy Sci.* 91, 4414-4423. doi:10.3168/jds.2007-0980
